# *Arbutus andrachne* Extracts Exhibit In Vitro Neuraminidase (N9) Inhibitory Activity: A Potential Herbal Strategy Against Avian Influenza

**DOI:** 10.3390/life16040560

**Published:** 2026-03-29

**Authors:** Areej Abuhammad, Fatma Afifi, Nour H. Aboalhaija, Mohammed H. Kailani, Mutasem O. Taha, Tamara Sabri, Zahra Fauri, Ismail Abaza

**Affiliations:** 1 Department of Pharmaceutical Sciences, School of Pharmacy, The University of Jordan, Amman 11942, Jordan; 2 Department of Pharmaceutical Chemistry and Pharmacognosy, Faculty of Pharmacy, Applied Science Private University, Amman 11931, Jordan; 3 Department of Pharmaceutical Sciences, Faculty of Pharmacy, Al-Zaytoonah University of Jordan, Amman 11733, Jordan; 4 Department of Chemistry, School of Science, The University of Jordan, Amman 11942, Jordan

**Keywords:** *Arbutus andrachne*, Ericaceae, neuraminidase inhibition, flavonoids, avian influenza, hyperoside

## Abstract

The rise in emerging viral outbreaks has intensified the need for novel antiviral therapies and highlighted the untapped potential of natural products. Influenza viruses, particularly avian strains, continue to evolve rapidly, yet the antiviral properties of Jordan’s native plants remain largely unexplored. This study focused on avian influenza and screened twelve endemic plant species, using ethanol to selectively extract polar phytochemicals likely to interact with the hydrophilic active site of neuraminidase (NA). Among these, *Arbutus andrachne* leaf and fruit extracts emerged as potent in vitro inhibitors of recombinant N9 neuraminidase, a key enzyme in influenza replication, with IC_50_ values of 31.6 µg/mL and 32.9 µg/mL, respectively. LC-MS analysis identified hyperoside as the major shared flavonoid in both extracts, which may contribute to the observed inhibitory activity. These findings support the potential of *A. andrachne* as a natural source for herbal preparations with antiviral activity.

## 1. Introduction

Since the late 20th century, viral outbreaks and pandemics such as bird flu, swine flu, SARS, and COVID-19 have intensified the global search for novel antiviral agents, highlighting the critical need to explore underutilized botanical resources [1,2]. These events have exposed significant vulnerabilities in public health systems globally and underscored the limitations of current antiviral medications, especially against rapidly mutating viruses [3]. In response, the World Health Organization (WHO) and other health authorities have stressed the urgent need for more adaptable and swiftly deployable treatments [4,5]. Influenza viruses continue to evolve and present public health challenges worldwide, reinforcing the importance of expanding antiviral research, particularly through the investigation of natural sources. Investigating the broad potential of indigenous plant extracts and plant-based compounds represents a promising strategy for identifying novel antivirals that could strengthen future pandemic preparedness and response.

The vast, untapped reservoir of plant-based compounds holds immense potential for developing novel antiviral agents [6]. For centuries, treatments derived from plants have proven effective against a wide range of pathogens, including those responsible for viral infections [7,8,9]. Recent scientific efforts have further validated the efficacy of various plant extracts and phytochemicals in fighting viral agents, demonstrating their potential within a modern pharmacological framework [8,10,11,12]. A notable example is shikimic acid, primarily sourced from Chinese star anise (*Illicium verum*), which is crucial in synthesizing oseltamivir (Tamiflu), a primary treatment for influenza [13,14]. This example highlights the vital role of plants in the pharmaceutical industry, particularly in addressing viral threats. Additionally, extracts from *Echinacea* spp. and *Silybum marianum* (milk thistle) have been shown to alleviate cold and flu symptoms and protect against hepatitis C, respectively [15]. The discovery of antiviral properties in compounds like saponins and flavonoids has opened new avenues for therapeutic applications against complex viruses [16,17,18,19]. Notably, robinetin has recently been discussed for its antiviral activity [20], and orientin has shown efficacy against Para 3 virus and *Herpes simplex* Virus Type 2 (HSV-2) [21,22,23]. Nevertheless, the full spectrum of plant biodiversity remains largely unexplored for antiviral drug discovery and development. This oversight underscores the critical need for further exploration into botanical resources, an endeavor that could reveal new strategies in combating viral diseases [24]. The rich history of botanical antivirals sets the stage for focused studies of specific ecosystems, such as the eastern Mediterranean, which may contain unique compounds with antiviral properties.

Jordan’s diverse topography and climate, situated at the crossroads of four bio-geographical regions, contribute to its rich biodiversity (Figure S1, Supplementary Information). This unique positioning has led to the presence of approximately 2900 plant species across 900 genera and 140 families [25,26]. Notably, about 20% of these plants are utilized in traditional medicine, highlighting their cultural and medicinal importance [27,28]. The extensive use of these plants, deeply rooted in the traditional practices of local communities, underscores a vast, yet largely untapped reservoir of botanical resources [29]. This traditional knowledge, combined with Jordan’s diverse flora, offers fertile ground for discovering novel antiviral agents. Locals, often guided by generations of herbalists, utilize these plants to treat a range of ailments, from the mundane to severe, life-threatening, or chronic diseases [29]. This widespread use, supported by empirical knowledge and anecdotal evidence, offers a unique opportunity for scientific validation and the discovery of novel antiviral agents. In this context, *Arbutus andrachne*, a native medicinal plant with a history of traditional use in the region, was selected for evaluation. As global challenges like influenza continue to evolve, the development of effective antiviral treatments becomes increasingly critical. Plant species such as *A. andrachne* are promising candidates for screening due to their phytochemical diversity and potential biological activity.

The critical role of neuraminidase (NA) in the viral replication cycle makes it a prime target for antiviral drugs, offering potential for both prophylactic and therapeutic applications [30]. Despite the existence of NA inhibitors like oseltamivir and zanamivir, the rapid mutation of influenza viruses continues to outpace current treatments, resulting in increased drug resistance [31]. This challenge is compounded by emerging avian strains, such as H7N9, which raise concerns regarding antiviral susceptibility, viral adaptation, and future treatment effectiveness. Avian influenza A (H7N9), first detected in humans in 2013, attracted major public health attention because of its association with severe respiratory disease, a high case fatality rate, and zoonotic transmission from poultry [32,33,34,35]. In addition, unlike highly pathogenic avian strains that cause severe disease in birds, H7N9 may circulate with limited clinical signs in avian hosts, raising concern about silent spread and continued reassortment with other avian influenza viruses [35,36]. These features, together with reported molecular signatures associated with mammalian adaptation, highlight the urgent need for novel inhibitors that can effectively address these evolving threats [32,37,38]. The development of such drugs is crucial not only for addressing immediate health concerns but also for enhancing our preparedness for future outbreaks.

Despite the urgent need, Jordan’s native flora remains largely unexplored as a source of NA inhibitors. Given the zoonotic potential of avian influenza viruses and growing concerns about antiviral resistance associated with avian influenza, this study focuses on N9 neuraminidase as a high-priority antiviral target. Jordan’s location along a major migratory bird corridor further supports the regional relevance of research on avian influenza-related targets [39]. Accordingly, this study evaluated the NA inhibitory potential of selected endemic plant species using in vitro biochemical assays. By exploring underutilized botanical resources, this research supports the broader effort to identify herbal sources of bioactive compounds with antiviral relevance.

## 2. Materials and Methods

### 2.1. Materials

Unless otherwise specified, chemicals and reagents used in this study were purchased from Sigma-Aldrich, Gillingham, UK. All chemicals were of analytical or molecular biology grade. Ethanol (analytical grade) was used for plant extraction. Solvents for LC-MS analysis, including methanol, acetonitrile, DMSO, and water, were LC-MS grade. A panel of 59 standard compounds used for metabolite profiling is listed in Supplementary Table S1. Enzyme assay reagents included MES buffer, CaCl_2_, Na_2_CO_3_, and DMSO. MUNANA (≥96.5% HPLC), 4-methylumbelliferone (≥98%), oseltamivir phosphate (>98%), hyperoside, and quercetin (≥95.0% HPLC) were used as received. Enzyme assays were conducted in black 96-well half-area microplates (Corning, NY, USA). Ultrapure water (type-1) was prepared in-house. Recombinant N9 neuraminidase from A/tern/Australia/G70C/75 (H11N9) and NB neuraminidase from B/Lee/40 were prepared as previously described [40,41].

### 2.2. Bioinformatics Analysis of N9 Neuraminidase Sequences

The amino acid sequences for NA from A/tern/Australia/G70C/75 (H11N9) and A/Shanghai/02/2013 (H7N9) were retrieved from the National Center for Biotechnology Information (NCBI) database (http://www.ncbi.nlm.nih.gov; accessed on 15 January 2024). The H11N9 sequence corresponded to the recombinant N9 neuraminidase used in the enzymatic assay, whereas H7N9 was included as a representative zoonotic avian influenza strain of broader public health relevance for sequence comparison. Sequence alignments were performed using Clustal Omega (http://www.ebi.ac.uk/Tools/msa/clustalo/; accessed on 15 January 2024). The resulting alignments were visualized with ESPript 2.2 (http://espript.ibcp.fr/ESPript/ESPript/; accessed on 15 January 2024) to highlight conserved residues and provide a clear comparison between the two NA sequences.

### 2.3. Plant Material and Sample Preparation

The analyzed plant samples were collected by the authors from various biogeographic zones in Jordan in 2023; the exact collection locations and voucher specimen numbers are provided in Figure S1 and Table S2 (Supplementary Information) [42,43,44]. All plant species studied are native and not classified under any conservation status; therefore, no collection permits were required. Samples were collected exclusively from public land, using only seasonally available aerial parts harvested at appropriate times. Roots and rhizomes were not disturbed, thus ensuring minimal impact on plant populations and their natural regeneration. The samples were morphologically authenticated by Barakat Abu Irmaileh and Fatma Afifi using descriptive references [26,27,45]. They were shed air-dried until reaching a constant weight, finely milled, and stored protected from light and moisture. Voucher specimens are stored in the Department of Pharmaceutical Sciences, School of Pharmacy, The University of Jordan, and are available upon request.

For extraction, 10 g of each plant sample was suspended in 100 mL of 70% ethanol, gently heated to near boiling, wrapped with parafilm, and left to soak overnight (~16 h) at ambient temperature to support efficient extraction. This extraction approach was selected to obtain reproducible crude ethanolic extracts suitable for initial biological screening while limiting extraction time. Subsequently, the extracts were filtered using grade 5 qualitative filter paper (Whatman, Maidstone, UK) under vacuum. The clear filtrate was subjected to solvent evaporation to dryness using a rotary evaporator (Buchi, Flawil, Switzerland). The resulting solid crude extracts were stored at 4 °C until analysis. For *A. andrachne*, extraction of 10 g of dried leaves and fruits yielded 207 mg and 223 mg of crude ethanol extract, respectively (~7–8% *w*/*w*). Stock solutions of all dried crude extracts were prepared at 30 mg/mL in DMSO. Before analysis in the in vitro NA inhibition assays, all sample solutions were filtered through a 0.45 μm syringe filter (Corning, NY, USA). After identification of promising NA inhibitory activity in the ethanolic extracts, aqueous extracts of *A. andrachne* leaves and fruits were prepared by substituting ethanol with water and applying the same extraction procedure. The resulting dried aqueous extracts were then prepared as stock solutions in the same manner and at the same concentration (30 mg/mL in DMSO).

### 2.4. Evaluation of Neuraminidase Inhibitory Activity

NA inhibitory activity was assessed using the fluorescence-based assay described previously [46]. In the primary screening step, 12 plant extracts were tested against recombinant N9 neuraminidase from A/tern/Australia/G70C/75 (H11N9), with oseltamivir included as the positive control at a final concentration of 1 µM. Extracts that showed activity in this initial screen were then further evaluated against recombinant NB neuraminidase from B/Lee/40 virus. In addition to the crude extracts, the commercially available flavonoids hyperoside and quercetin were evaluated in the NA inhibition assay as reference flavonoids [47,48,49]. Details of the recombinant enzymes used in this study are provided in Section 2.1 (Materials) and in the original reports describing their preparation [40,41].

Enzyme dilutions were optimized in assay buffer (20 mM MES, pH 6.5, and 4 mM CaCl_2_) to maintain uninhibited reactions within the linear response range of the assay. Each plant extract (1 µL of 30 mg/mL in DMSO) or hyperoside/quercetin (1 µL of 30 mM in DMSO) was mixed with 14 µL of diluted enzyme and preincubated for 30 min at 22 °C in black half-area 96-well plates. The reactions were initiated by adding 15 µL of MUNANA substrate prepared in assay buffer, giving a final substrate concentration of 5 µM, a final extract concentration of 1000 µg/mL, and a final hyperoside/quercetin concentration of 1000 µM in a total reaction volume of 30 µL. Reactions were incubated at 22 °C in the dark for 20 min for N9 neuraminidase or 15 min for NB neuraminidase and were then terminated by adding 60 µL of 50 mM Na_2_CO_3_ (pH 10.0). Fluorescence was measured at an excitation wavelength (λ_ex) of 365 nm and an emission wavelength (λ_em) of 460 nm using an Infinite 200 PRO M Plex plate reader (Tecan, Grödig, Austria). Each assay plate included an uninhibited enzyme control, oseltamivir as the positive control (with a final concentration of 1 µM), and extract-specific fluorescence attenuation controls prepared in the absence of an enzyme to ensure consistent interpretation of inhibitory activity. Fluorescence values from attenuation controls were subtracted from the corresponding sample readings before the calculation of percentage inhibition. Signals from uninhibited reactions were typically 40,000–60,000 fluorescence units and remained within the linear range of the assay. Enzyme activity was determined from the rate of 4-methylumbelliferone formation, and its concentration was calculated using a standard curve generated from serial dilutions of 4-methylumbelliferone. All experiments were performed in triplicate, and the results are presented as mean ± SD from three independent measurements. IC_50_ values were determined using 10 two-fold serial dilutions of each extract (1.95–1000 µg/mL) or hyperoside (1.95–1000 µM), and the data were fitted to a three-parameter nonlinear sigmoidal dose–response model using GraphPad Prism (version 10.0). Percent enzyme inhibition was calculated from the mean fluorescence values after correction for attenuation controls.

### 2.5. Analysis of the Chemical Composition of A. andrachne Fruit and Leaf Extracts by LC-MS

The samples of active extracts (ethanol extracts of *A. andrachne* leaves and fruits) were analyzed by liquid chromatography coupled to mass spectrometry (LC-MS), using a Bruker Daltonik Impact II ESI-Q-TOF system equipped with a Bruker Daltonik Elute UPLC system (Bruker Daltonik, Bremen, Germany). This methodology was adapted for the identification and quantification of secondary metabolites in plant extracts [50]. All solvents used for LC-MS analysis, including acetonitrile, methanol, water, and formic acid, were LC/MS grade. Standard solutions used for metabolite identification were prepared by dissolving the appropriate amount of each compound in dimethyl sulfoxide (DMSO, analytical grade), followed by dilution with acetonitrile. The analysis was performed using a high-resolution Bruker TOF MS with an Ion Source Apollo II ion Funnel electrospray source. The operational parameters were set as follows: a capillary voltage of 2500 V, a nebulizer gas pressure of 2.0 bar, a dry gas (nitrogen) flow rate of 8 L/min, and a dry temperature of 200 °C. The mass accuracy was maintained at ˂1 ppm, with a mass resolution of 50000 FSR (Full-Sensitivity Resolution) and a TOF repetition rate of up to 20 kHz. Chromatographic separation was achieved using a Bruker solo 2.0_C-18 UHPLC column (100 mm × 2.1 mm × 2.0 μm) at a flow rate of 0.51 mL/min and a column temperature of 40 °C. The mobile phase consisted of two solvents: solvent A (water with 0.05% formic acid) and solvent B (acetonitrile). The gradient program was as follows: a linear gradient from 5% to 80% B over 0–27 min, followed by 95% B from 27 to 29 min, and a return to 5% B at 29.1 min. The total analysis time was 35 min in both positive and negative ion modes. The injection volume for each sample was 3 μL. Stock solutions were prepared by dissolving each extract sample in 2.0 mL DMSO, then diluted to 50 mL with acetonitrile. After centrifugation at 4000 rpm for 2 min, 1.0 mL of each sample was transferred to the autosampler, and 3.0 μL was injected into the LC-MS system. The identification of chemical composition was performed by matching the exact *m*/*z* values and retention times with those of authentic standards analyzed under the same chromatographic conditions. Peak areas were calculated and used to quantify the relative concentrations of the detected compounds. Each sample was analyzed in duplicate.

## 3. Results

**Figure 1:**
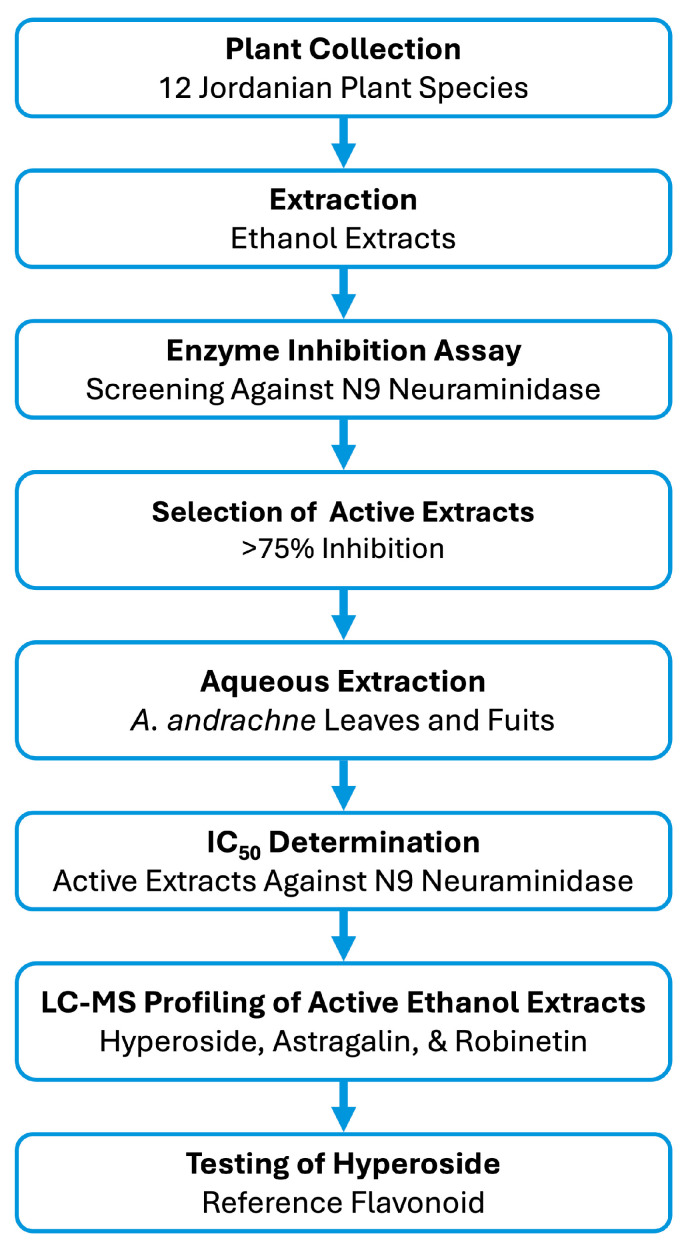
Experimental workflow of the study. Ethanolic extracts of twelve Jordanian plant species were screened for inhibitory activity against recombinant N9 neuraminidase using a fluorescence-based assay. Extracts showing >75% inhibition were selected for further evaluation, including preparation of aqueous and ethanolic leaf and fruit extracts of *A. andrachne*, IC_50_ determination, and LC-MS profiling of the active ethanolic extracts. Hyperoside and quercetin were also evaluated as reference flavonoids.

The overall experimental workflow is summarized in Figure 1. To identify herbal extracts with NA inhibitory activity, ethanolic extracts from twelve Jordanian plant species were first screened. Following identification of strong activity in *A. andrachne*, additional ethanolic and aqueous leaf and fruit extracts of this species were prepared and evaluated. To support the relevance of the biochemical screening model, the NA sequence from A/tern/Australia/G70C/75 (H11N9) was compared with that of A/Shanghai/02/2013 (H7N9), a zoonotic avian influenza strain of broader public health relevance, revealing 93.1% sequence identity and supporting the suitability of H11N9 as a screening model (Figure S2). NA inhibition was first assessed using an in vitro fluorescence-based assay, followed by IC_50_ determination of active *A. andrachne* extracts. Finally, the phytochemical composition of the active ethanolic extracts was analyzed by LC-MS to explore flavonoid constituents potentially contributing to the observed activity.

### 3.1. Strong NA Inhibitory Activity of A. andrachne Leaf and Fruit Extracts

Twelve ethanol extracts from selected Jordanian plants were screened for NA inhibitory activity using a fluorescence-based enzyme assay. Ethanol (70% *v*/*v*) was selected as the extraction solvent to enrich polar phytochemicals, guided by a previous analysis of the hydrophilic nature of the NA active site [51]. Among the tested samples, the ethanol extract of *A. andrachne* leaves exhibited the strongest inhibitory effect, achieving 94.8% inhibition at 1000 µg/mL (Table 1). The fruit extract also demonstrated high inhibition, reaching 96.5% at the same concentration. Oseltamivir, a standard neuraminidase inhibitor included as a positive control, completely inhibited N9 neuraminidase at 1 µM under the assay conditions used, while the reference flavonoids hyperoside and quercetin showed 91.6% and 71.9% inhibition, respectively (Table 2). These results confirmed the expected responsiveness of the assay system. The extract yield (7–8% *w*/*w*) indicated that bioactive material can be obtained efficiently from modest quantities of plant material. These findings suggest that both the leaves and fruits of *A. andrachne*, traditionally collected for medicinal purposes, contain constituents with strong in vitro NA inhibitory activity and may warrant further exploration within the context of herbal antiviral research.

### 3.2. Ethanol Extracts Exhibited Higher NA Inhibitory Activity than Aqueous Extracts

Following the initial screening, additional extracts of *A. andrachne* were prepared from both leaves and fruits using ethanol and water as solvents. All extracts were tested at a fixed concentration of 1000 µg/mL against avian N9 neuraminidase. The ethanol extract of the fruits exhibited the highest inhibition (96.5%), followed closely by the ethanol leaf extract (94.8%) (Table 3). The aqueous leaf extract showed moderate inhibition (78.4%), whereas the aqueous fruit extract displayed lower activity (41.6%). These results indicate that ethanol extracts exhibited higher NA inhibitory activity than aqueous extracts, likely due to greater extraction efficiency of flavonoids and other polyphenolic compounds in ethanol. The findings also reflect the influence of both plant part and solvent on extract composition and bioactivity. As both leaves and fruits are traditionally used in herbal preparations, these outcomes support the relevance of ethanolic extracts of *A. andrachne* for further investigation in herbal medicine contexts.

### 3.3. Extracts Retain Activity Against Influenza B Neuraminidase

The ethanol and aqueous extracts of *A. andrachne* were further tested against NB neuraminidase from influenza B/Lee/40, which possesses a structurally distinct active site and is commonly used as a stringent control to assess broad-spectrum inhibition. The ethanol fruit and leaf extracts retained substantial inhibitory activity against NB neuraminidase, showing 65.2% and 58.7% inhibition, respectively (Table 3). The aqueous leaf extract showed reduced inhibition (23.5%), and the aqueous fruit extract was not tested against NB neuraminidase.

The decision to include NB neuraminidase in this study was based on its well-documented structural divergence from N9 neuraminidase. As such, NB neuraminidase provides a conservative test for assessing whether extract activity extends beyond subtype-specific inhibition. While neuraminidase subtypes such as N1 and N2 are of clear clinical relevance, their inclusion requires additional recombinant systems and was beyond the scope of this initial extract screening effort.

Nonetheless, the observed inhibition of NB neuraminidase by both ethanol extracts suggests that the active constituents may interact with conserved features across NA subtypes, supporting their potential as broad-spectrum agents within herbal antiviral research.

### 3.4. Ethanol Extracts Show Dose-Dependent Inhibition with Low IC_50_ Values

**Figure 2:**
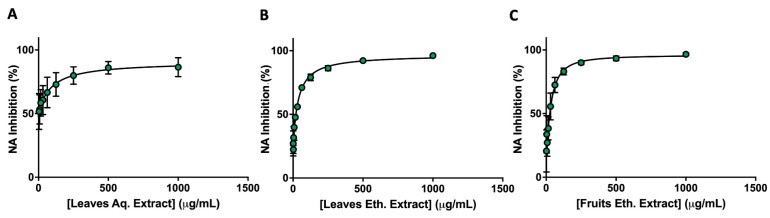
Inhibitory effects of *A. andrachne* extracts on N9 neuraminidase. Concentration-response curves for (**A**) aqueous (Aq.) leaf, (**B**) ethanol (Eth.) leaf, and (**C**) ethanol fruit extracts of *A. andrachne*. NA activity was evaluated as described in Methods, using 10 two-fold serial dilutions of each extract (1.95–1000 µg/mL) in triplicate. Percentage NA inhibition was calculated compared to the uninhibited control and plotted against extract concentration to determine the IC_50_. Data are presented as mean % inhibition ± SD of three independent experiments. The error bars are within the symbols.

To quantify the inhibitory potency of *A. andrachne* extracts, IC_50_ values were determined for the ethanol leaf and fruit extracts, as well as for the aqueous leaf extract. Concentration-dependent inhibition was observed for all tested samples. The ethanol leaf extract exhibited an IC_50_ of 31.6 µg/mL, while the fruit extract showed a comparable IC_50_ of 32.9 µg/mL (Table 3 and Figure 2). In contrast, the aqueous leaf extract demonstrated a higher IC_50_ of 158 µg/mL, indicating lower potency.

These values confirm the strong in vitro activity of the ethanol extracts and further support the selection of ethanol as a suitable solvent for extracting bioactive constituents. Given the traditional use of both plant parts in regional herbal medicine, the low IC_50_ values observed in this study highlight the potential utility of these extracts in future herbal formulations aimed at managing viral infections.

### 3.5. LC-MS Analysis Reveals Flavonoid-Rich Composition of Active Extracts

The active ethanol extracts of *A. andrachne* leaves and fruits were analyzed by LC-MS to identify their chemical composition. A total of 32 and 33 compounds were detected in the leaf and fruit extracts, respectively (Table S3; Figures S3 and S4). Flavonoids accounted for 55.75% of the total compounds in the leaf extract and 82.76% in the fruit extract.

In the ethanol leaf extract, robinetin (24.05%) and hyperoside (17.23%) were the major flavonoids, while 4-methylumbelliferone (21.32%) was the most abundant coumarin derivative. Minor flavonoids included orientin (8.27%) and 7,3′,4′,5′-tetrahydroxyflavone (4.45%). In contrast, the ethanol fruit extract was dominated by astragalin (35.61%) and hyperoside (29.65%), with robinetin present only in trace amounts (0.15%).

**Figure 3:**
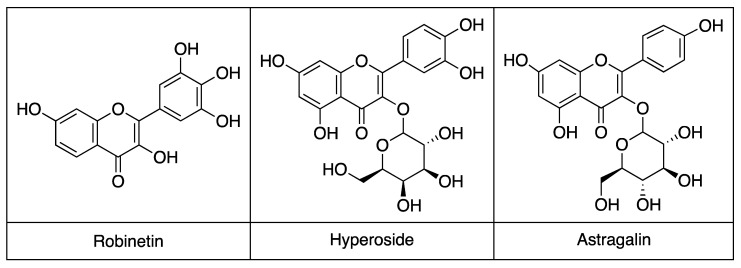
Chemical structures of the major flavonoids identified in the ethanol extracts of *A. andrachne*.

These results indicate that both extracts are rich in flavonoid glycosides, which are known to contribute to antiviral and antioxidant effects in herbal preparations. The major flavonoids identified in the active extracts share a closely related flavonol scaffold. Hyperoside and astragalin are structurally similar glycosides and differ mainly by one additional hydroxyl group on the B ring of hyperoside, whereas robinetin is a structurally related aglycone flavonol with a more highly hydroxylated B ring (Figure 3). Among the identified constituents, hyperoside, which was used as a reference flavonoid in this study, was further evaluated to determine its IC_50_, whereas robinetin, astragalin, and the other detected compounds were characterized by LC-MS only and were not individually tested, as the focus of this work was the profiling and activity evaluation of the whole plant extracts.

### 3.6. Hyperoside Showed Concentration-Dependent Inhibition of N9 Neuraminidase

**Figure 4:**
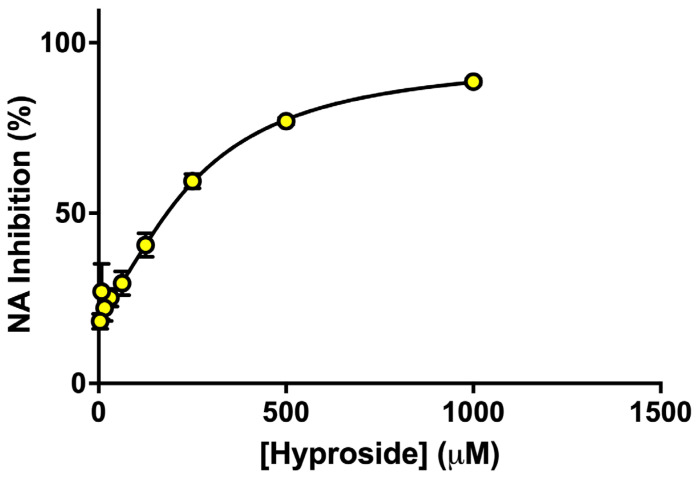
Hyperoside inhibition of N9 neuraminidase. Concentration response curve was determined using 10 two-fold serial dilutions of hyperoside (1.95–1000 µM). NA activity was evaluated as described in Methods at each concentration in triplicate. Percentage NA inhibition was calculated compared to the uninhibited control and plotted against hyperoside concentration to determine the IC_50_. Data are presented as mean % inhibition ± SD of three independent experiments. The error bars are within the symbols.

Among the reference flavonoids tested, hyperoside was selected for further evaluation against N9 neuraminidase because it was identified as a major flavonoid present in both active extracts. Hyperoside inhibited N9 neuraminidase in a dose-dependent manner, with an IC_50_ of 257.4 µM, equivalent to approximately 119.5 µg/mL based on its molecular weight (Figure 4). Its potency was lower than that of the crude ethanol extracts (IC_50_ = 31.6–32.9 µg/mL), indicating that hyperoside alone does not fully explain the extract activity.

## 4. Discussion

The continuous emergence of new influenza strains emphasizes the critical need to identify novel antiviral agents from untapped natural resources. This study aimed to identify bioactive herbal therapies or plant extracts, utilizing Jordanian flora as a promising untapped resource for discovering inhibitors of neuraminidase, a key enzyme in influenza replication. Among the tested plant extracts, *A. andrachne* leaves and fruits showed significant NA inhibition, making this vulnerable species a promising candidate for antiviral development. Commonly known as the Eastern Strawberry or Grecian Strawberry, *A. andrachne* is native to the Levant, Mediterranean region, and North Africa, with a history of traditional medicinal use in countries such as Jordan, Lebanon, and Turkey [45,52,53,54]. Although it is not traditionally used for treating viral respiratory infections, it has been employed in folk medicine for gastrointestinal, anti-inflammatory, and general health purposes, which aligns with its observed flavonoid content and antioxidant activity. To the best of our knowledge, this is the first study to report NA inhibitory activities for *A. andrachne*. These findings underscore the potential of this plant as a natural source of compounds with potential relevance to avian influenza.

These findings reveal that crude ethanol extracts of *A. andrachne* exhibited substantial NA inhibitory activity, comparable to previously reported plant-based antiviral agents. Consistent with studies on plant extracts with antimicrobial and antioxidant properties, crude extracts often demonstrate enhanced activity compared to isolated secondary metabolites [55,56]. Kim, Li, Kim, Chung and Choi [55] showed that crude extracts with similar NA inhibitory levels effectively suppressed viral replication in vitro and in vivo, which aligns with our findings. However, this study was conducted solely using in vitro enzyme assays, and no cellular or in vivo models were included. This suggests that *A. andrachne* extracts may offer antiviral efficacy at the biochemical level, though additional studies are needed to explore their biological relevance.

LC-MS analysis revealed a diverse flavonoid profile in *A. andrachne* extracts, including robinetin, astragalin, hyperoside, and coumarin derivatives such as 4-methylumbelliferone. Notably, the flavonoid content of the fruits surpassed previous reports, potentially due to geographic, environmental, or methodological factors [57,58,59]. Because the principal aim of the present study was to identify the biological activity of whole plant extracts against influenza neuraminidase, its main contribution lies in demonstrating that *A. andrachne* leaf and fruit extracts represent a previously unreported flavonoid-rich source of NA inhibitory activity at the whole-extract level. More detailed characterization of the individual secondary metabolites was beyond the scope of the present work. Among the major flavonoids identified in the active extracts, hyperoside was of particular interest because it was present in both the leaf and fruit ethanol extracts and showed substantial inhibitory activity against N9 neuraminidase. In this context, hyperoside was used as a relevant reference flavonoid for contextualizing the observed extract activity. Astragalin and robinetin were likewise notable, as they were among the dominant flavonoids detected in the fruit and leaf extracts, respectively. By contrast, quercetin, although active in the enzymatic assay, was detected only in trace amounts in the ethanol fruit extract and was not detected in the ethanol leaf extract. Previous studies have reported hyperoside’s moderate NA inhibitory activity against N1 and N2 strains, although it exhibits lower activity against N9 [47,48]. Astragalin has been previously documented to possess NA inhibitory activity [60,61], whereas the possible contribution of robinetin in the present study was not directly evaluated and is instead suggested by its abundance in the active leaf extract and its structural similarity to flavonoids with known neuraminidase inhibitory activity. While LC-MS analysis identified multiple candidate metabolites, the exact contribution of each compound to the overall activity remains to be experimentally validated. These findings indicate that multiple secondary metabolites may contribute to the observed extract activity; however, the nature and extent of their individual or combined effects remain to be determined experimentally.

The observation that crude extracts exhibited comparable or superior activity to individual secondary metabolites highlights the potential role of multiple contributing constituents within the extract. Previous studies have attributed this phenomenon to factors such as enhanced solubility, stability, or complementary pharmacodynamic effects within the extract [62]. However, additive or synergistic interactions among these compounds were not directly evaluated in the present study and therefore remain to be confirmed experimentally. Thus, the present data support the possibility of combined effects, but do not establish a specific synergistic mechanism. Although N1 and N2 subtypes were not included in this study, neuraminidase from influenza B (NB), which possesses a structurally distinct active site, was selected as a stringent specificity control to assess broader inhibitory potential beyond the avian N9 subtype. Given the clinical importance of N1 and N2 subtypes, as well as known oseltamivir-resistant variants such as H274Y and R292K, future testing against these targets would be valuable to further evaluate the scope of bioactivity. However, the current study is limited to biochemical screening and does not assess cell-based activity, bioavailability, or therapeutic potential.

The presence of flavonoids such as robinetin and orientin, known for their pharmacological properties [20,63,64,65], may contribute not only to NA inhibition but also to broader therapeutic effects. While these results are promising, this study is limited by its in vitro design, lack of cell-based antiviral assays, and absence of bioassay-guided fractionation. Because the extraction protocol involved gentle heating in 70% ethanol, the resulting phytochemical profile may differ from that obtained by cold maceration, and future comparisons of extraction methods may help define the contribution of heat-sensitive constituents more fully. The observed NA inhibitory activity may therefore reflect the contribution of multiple constituents, but the individual roles of these compounds remain unresolved. Further work is needed to isolate and validate the active constituents and to determine whether their effects are additive, synergistic, or independent. Additionally, factors such as cellular uptake, metabolic stability, and selectivity were not evaluated and are beyond the current scope. Optimizing extraction protocols and sustainable harvesting practices will be crucial for developing these findings into accessible, plant-based antiviral therapies, especially considering the ecological vulnerability of *A. andrachne*.

These findings align with traditional uses of *A. andrachne* and contribute to the broader understanding of flavonoid-rich plants as antiviral candidates within herbal medicine. In summary, this study demonstrates that ethanol extracts of *A. andrachne* leaves and fruits exhibit promising in vitro inhibitory activity against avian neuraminidase (N9), likely due to their rich flavonoid content. Hyperoside, together with other major identified flavonoids such as robinetin and astragalin, may contribute to the observed activity, although their individual roles and any combined effects were not directly resolved in the present study. These findings suggest that *A. andrachne* may serve as a valuable natural source for further exploration of neuraminidase inhibitors within the scope of herbal medicine, particularly as a flavonoid-rich extract with measurable activity at the whole-extract level.

## 5. Conclusions

This study addressed the limited exploration of Jordanian medicinal plants as sources of influenza neuraminidase inhibitors by evaluating the activity of selected endemic species against recombinant N9 neuraminidase. Among the screened plants, the ethanolic leaf and fruit extracts of *A. andrachne* showed the strongest inhibitory activity, with comparable potency at the extract level. LC-MS profiling revealed that the active extracts were rich in flavonoids, particularly hyperoside, robinetin, and astragalin, while further testing identified hyperoside as a contributing flavonoid but not the sole determinant of the observed activity.

Taken together, these findings identify *A. andrachne* as a previously unreported flavonoid-rich source of neuraminidase inhibitory activity at the whole-extract level. Although the present work was limited to biochemical screening and did not resolve the individual roles of all detected metabolites, it provides a strong basis for future studies on bioactive extract standardization, fractionation, and antiviral validation in cellular systems. These results support the potential of *A. andrachne* as a promising herbal source for further investigation in antiviral research.

## Figures and Tables

**Table 1:** Inhibitory effects of ethanol extracts from selected plant species on N9 neuraminidase.

Plant Family	Plant Name	Part Used	NA Inhibition (%)
Araceae	*Arum palaestinum*	Aerial	11.76 ± 9.9
	*Biarum angustatum*	Leaves	31.17 ± 5.9
	*Eminium spiculatum*	Leaves	−8.7 ± 2.6
Asteraceae	*Artemisia vulgaris*	Aerial	50.98 ± 1.13
	*Santolina chamaecyperissus*	Aerial	57.39 ± 0.0
Capparaceae	*Capparis spinosa*	Fruits	−26.18 ± 11.1
Caprifoliaceae	*Scabiosa argentea*	Aerial	28.91 ± 9.4
Caryophyllaceae	*Silene arabica*	Aerial	−6.55 ± 8.5
Ericaceae	*Arbutus andrachne*	Leaves	94.80 ± 1.4
Fabaceae	*Parkinsonia aculeata*	Aerial	16.97 ± 1.1
Lamiaceae	*Sideritis cretica*	Aerial	36.96 ± 3.5
Salvadoraceae	*Salvadora persica*	Leaves	−92.18 ± 16.2

**Table 2:** Chemical structures and inhibitory activity of oseltamivir as the positive control and selected reference flavonoids against N9 neuraminidase ^a^.

Name	Structure	Inhibition (%)
Oseltamivir	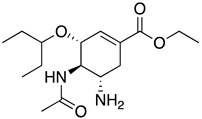	100.3 ± 0.3
Hyperoside	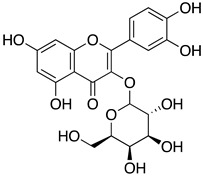	91.6 ± 2.9
Quercetin	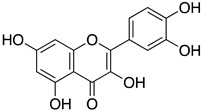	71.9 ± 5.7

^a^ Oseltamivir was tested at 1 µM; hyperoside and quercetin were tested at 1000 µM.

**Table 3:** Inhibitory activity of *A. andrachne* leaf and fruit extracts on N9 and NB neuraminidases.

		NA Inhibition (%) ^a^		N9 IC_50_ (µg/mL)
		N9	NB	
Leaves	Aqueous	78.4 ± 4.8	23.5 ± 8	158 ± 25 ^b^
	Ethanol	94.8 ± 1.4	58.7 ± 1.7	31.6 ± 7.1
Fruits	Aqueous	41.6 ± 8	NT ^c^	NT ^c^
	Ethanol	96.5 ± 0.1	65.2 ± 1	32.9 ± 4.0

^a^ Extract tested at 1000 µg/mL final concentration in the assay solution. ^b^ Apparent IC_50_. ^c^ NT, not tested.

## Data Availability

The datasets used and/or analyzed during the current study are available from the corresponding author on reasonable request.

## Supplementary Materials

The following supporting information can be downloaded at: https://www.mdpi.com/article/10.3390/life16040560/s1, Table S1: Standards used for identification 1 of *m*/*z* and retention time; Table S2: Detailed information on plant samples used in this study; Table S3: Chemical composition of ethanol extracts from *A. andrachne* leaves and fruits via LC-MS analysis; Figure S1: Biogeographic zones of Jordan; Figure S2: Amino acid sequence alignment of NA from H11N9 and H7N9 viruses; Figure S3: LC-MS chromatograms of *A. andrachne* leaf extracts and identified compounds; Figure S4: LC-MS chromatograms of *A. andrachne* fruit extracts.
