# Supplementary material for: Arbutus andrachne Extracts Exhibit In Vitro Neuraminidase (N9) Inhibitory Activity: A Potential Herbal Strategy Against Avian Influenza
Source: Life (Basel). 2026 Mar 29;16(4):560. doi: 10.3390/life16040560 (PMC13117706; doi:10.3390/life16040560)

Supplementary Information

***Arbutus andrachne* Extracts as Novel Inhibitors of Avian Neuraminidase N9**

Running Head: ***A. andrachne Inhibits NA***

Areej Abuhammad[1,*], Fatma U. Afifi[1,2], Nour Aboalhaija[3], Mutasem Taha[1], Mohammed H. Kailani[4], Tamara Sabri[1], Zahra Fauri[4], Ismail Abaza[1]

[1]Department of Pharmaceutical Sciences, School of Pharmacy, The University of Jordan, Amman, Jordan.

[2]Department of Pharmaceutical Chemistry and Pharmacognosy, Faculty of Pharmacy, Applied Science Private University, Amman, Jordan

[3]Department of Pharmaceutical Sciences, Faculty of Pharmacy, Al-Zaytoonah University of Jordan, Amman, Jordan

[4]Department of Chemistry, School of Science, The University of Jordan, Amman, Jordan.

*Address correspondence to: Areej Abuhammad, School of Pharmacy, University of Jordan, Queen Rania St. Amman 11942 Jordan, ORCID ID: 0000-0003-4978-5059

Email: a.abuhammad@ju.edu.jo

Table S1: Standards used for identification of m/z and retention time.

No	Analyte Name
1	3-Oxocostusic acid
2	4-Hydroxybenzoic acid
3	4-Hydroxy-coumarin
4	4-OH-coumarinic acid
5	5,6,4'-Trihydroxy-7,3'-dimethoxyflavone
6	7-Hydroxy-coumarin
7	Adenosine
8	Aesculetin
9	Ajugoside
10	Alpha amyrin
11	Apigenin
12	Benzoic acid
13	Beta-amyrin
14	Caffeic acid phenethyl ester (cape)
15	Caffeic acid
16	Caffeine
17	Carnosic acid
18	Catechin
19	Catechol
20	Chlorogenic acid
21	Chrysin
22	Cinnamic acid
23	Colchicine
24	Crotonoyl cosmosiin
25	Ellagic acid
26	Epicatechin
27	Ferulic acid
28	Galangin
29	Galangustin
30	Gallic acid
31	Gallic acid-ethyl
32	Hesperidin
33	Hispidulin
34	Hyperoside
35	Isoorientin
36	Kaempferol
37	Kumatakenin
38	Ladanetin
39	Lupeol

40	Luteolin
41	Luteolin 7-O-glucoside
42	Myricetin
43	Naringenin
44	Naringin
45	p-Coumaric acid
46	Pivalic acid
47	Procayadnin
48	Quercetin
49	Quercetin 3,3'-dimethyl ether
50	Resveratrol
51	Rosmarinic acid
52	Rutin
53	Salvianolic acid B
54	Succinic acid
55	Syringic acid
56	Taraxasterol
57	Vanillic acid
58	Vanillin
59	Vitexin

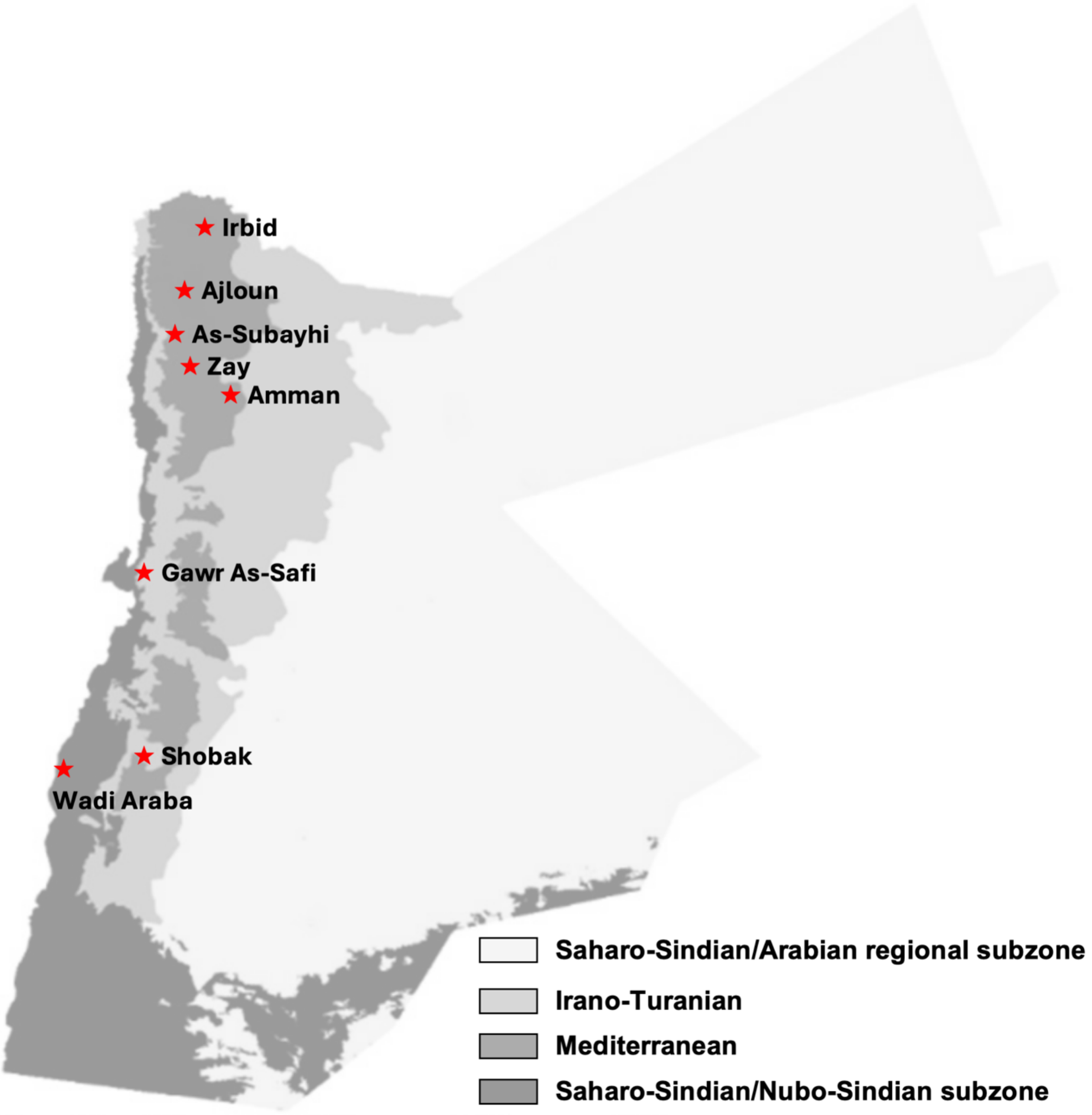

Figure S1: Biogeographic Zones of Jordan

This map highlights the four principal bioclimatic or biogeographical regions recognized in Jordan: Mediterranean, Irano-Turanian, Saharo-Arabian, and Sudanian (representing tropical penetration). It also marks the specific areas and locations from which plant samples were collected. The map underscores the ecological diversity of Jordan and is essential for contextualizing the collection sites of plant samples used in this study. Map modified based on [43].

Table S2: Detailed information on plant samples used in this study*.

Plant Name (Part Used)	Voucher ID	Collection Location	Harvest Time	Biogeographic Zone	Geographic Coordinates
A. palaestinum (aerial)	FMJ-ARAC-13	Irbid	March 2023	Mediterranean	32.5644° N, 35.8499° E
B. angustatum (leaves)	FMJ-ARAC-24	Irbid	March 2023	Mediterranean	32.5644° N, 35.8499° E
E. spiculatum (leaves)	FMJ-ARAC-31	Irbid	March 2023	Mediterranean	32.5644° N, 35.8499° E
A. vulgaris (aerial)	FMJ-ASTE-96	Shobak	May 2023	Irano-Turanian	30.5179° N, 35.5571° E
S. chamaecyperissus (aerial)	FMJ-ASTE-981	Amman	June 2023	Mediterranean	31.9544° N, 35.9106° E
C. spinosa (fruits)	FMJ-CAPP-17	Amman	August 2023	Mediterranean	31.9544° N, 35.9106° E
S. argentea (aerial)	FMJ-CAPR-31	As-Subayhi	April 2023	Mediterranean	32.1557° N, 35.7215° E
S. arabica (aerial)	FMJ-CARY-244	Ajloun	April 2023	Mediterranean	32.3276° N, 35.7518° E
A. andrachne (leaves)	FMJ-ERIC-11	Zay	April 2023	Mediterranean	32.1066° N, 35.7083° E
A. andrachne (fruits)	FMJ-ERIC-12	Zay	May 2023	Mediterranean	32.1066° N, 35.7083° E
P. aculeata (aerial)	FMJ-FABA-461	Ghawr As-Safi	May 2023	Sudanian Penetration	31.0380° N, 35.4873° E
S. cretica (aerial)	FMJ-LAMI-234	Ajloun	April 2023	Mediterranean	32.3276° N, 35.7518° E
S. persica (leaves)	FMJ-SALV-11	Wadi Araba	April 2023	Saharo-Arabian	29.6825° N, 35.2070° E

*Refer to Figure S1 for the biogeographic zones map of Jordan.

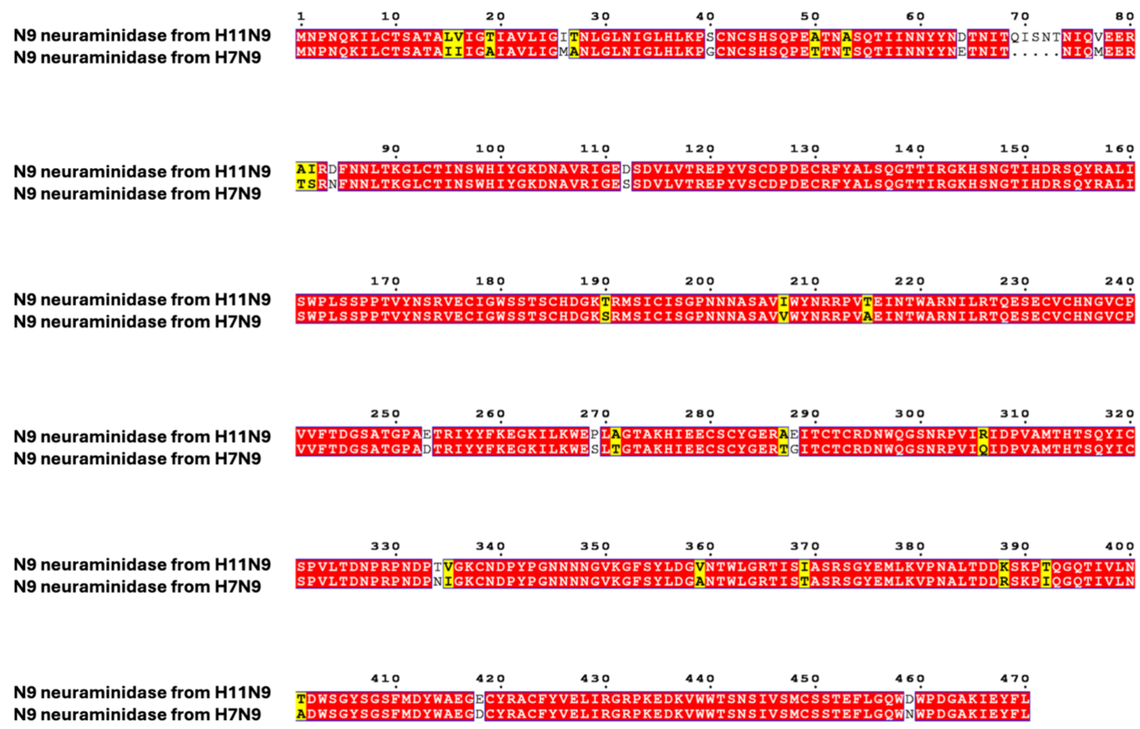

Figure S2: Amino Acid Sequence Alignment of NA from H11N9 and H7N9 Viruses. NA sequences from A/Tern/Australia/G70C/1975 (H11N9) and A/Shanghai/02/2013 (H7N9) are aligned using Clustal Omega. Conserved residues are highlighted in red, and similar residues in bold on a yellow background per the Risler matrix in ESPript. Dashes indicate sequence deletions, highlighting the length difference between the two strains. Figure prepared with ESPript 2.2.

Table S3: Chemical composition of ethanol extracts from *A. andrachne* leaves and fruits via LC-MS analysis.

No.	Compound Name	Formula	Molecular Weight	Retention Time (min)	Relative Abundance (%)	
					Leaves	Fruits
**1**	Succinic acid	$C_4H_6O_4$	117.0194	0.98	-	0.10
**2**	Gallic acid	$C_7H_6O_5$	169.01438	1.13	-	0.30
**3**	Protocatechuic aldehyde	$C_7H_6O_3$	137.02446	1.25	-	0.30
**4**	Gallocatechin	$C_{15}H_{14}O_7$	306.0738	1.66	-	2.60
**5**	Chlorogenic acid	$C_{16}H_{18}O_9$	354.0955	2.04	-	0.01
**6**	2,5-Dihydroxybenzoic acid	$C_7H_6O_4$	154.0267	2.14	0.23	0.06
**7**	Procyanidin B1	$C_{30}H_{26}O_{12}$	577.135	2.51	-	1.30
**8**	Arbutin	$C_{12}H_{16}O_7$	294.0717	2.82	8.13	-
**9**	Catechin	$C_{15}H_{14}O_6$	290.0789	2.85	-	9.00
**10**	Catechol	$C_6H_6O_2$	110.0368	3.04	1.16	-
**11**	4-Hydroxybenzoic acid	$C_7H_6O_3$	138.0312	3.10	0.97	-
**12**	Caffeic acid	$C_9H_8O_4$	180.0426	3.36	0.30	0.08
**13**	2,3-*trans*-3,4-trans-Leucocyanidin	$C_{15}H_{14}O_7$	306.077	3.54	-	0.01
**14**	Epicatechin	$C_{15}H_{14}O_6$	290.0792	3.81	2.67	-
**15**	Umbelliferone	$C_9H_{10}O_5$	162.0316	3.81	2.61	-
**16**	Epigallocatechin gallate	$C_{22}H_{18}O_{11}$	457.078	3.87	-	0.50
**17**	Vanillic acid	$C_8H_8O_4$	168.0416	4.07	0.71	-
**18**	Luteolin-7,3'-di-O-glucoside	$C_{27}H_{30}O_{16}$	610.154	4.30	-	0.02

**19**	Anthranilic acid	$C_7H_7NO_2$	137.0482	4.38	0.12	-
**20**	3-Glu-3,4',7-trihydroxyisoflavanone (NMR)	$C_{21}H_{22}O_{10}$	435.1283	4.47	0.20	-
**21**	Ethyl gallate	$C_9H_{10}O_5$	198.0531	4.54	0.51	0.80
**22**	Scopoletin	$C_{10}H_8O_4$	192.0424	4.62	0.30	-
**23**	Ferulic acid (trans)	$C_{10}H_{10}O_4$	194.0578	4.63	0.14	-
**24**	Hyperoside	$C_{21}H_{20}O_{12}$	464.0957	4.81	17.23	29.65
**25**	Isoorientin	$C_{21}H_{20}O_{11}$	448.1007	4.87	-	0.42
**26**	Scutellarein-7-glucuronide	$C_{21}H_{18}O_{12}$	462.0799	4.88	0.33	-
**27**	p-Coumaric acid	$C_9H_8O_3$	164.0472	4.9	0.32	-
**28**	3,7,3',4',5'-Pentahydroxyflavone (Robinetin)	$C_{15}H_{10}O_7$	302.0427	5.13	24.05	0.15
**29**	Saponarin	$C_{27}H_{30}O_{15}$	594.1607	5.13	0.14	0.13
**30**	Orientin	$C_{21}H_{20}O_{11}$	448.1007	5.15	8.27	-
**31**	7,3',4',5'-Tetrahydroxyflavone	$C_{15}H_{10}O_6$	286.0476	5.18	4.45	-
**32**	Taxifolin (3,3',4',5,7-pentahydroxylflavanone)	$C_{15}H_{12}O_7$	304.0581	5.21	0.11	-
**33**	Catechin gallate	$C_{22}H_{18}O_{10}$	442.0897	5.37	-	1.80
**34**	3,5-Dimethoxy-4-hydroxyacetophenone	$C_{10}H_{12}O_4$	196.0717	5.41	0.50	-
**35**	Rutin	$C_{27}H_{30}O_{16}$	610.1529	5.59	-	2.04
**36**	Vitexin	$C_{21}H_{20}O_{10}$	432.1049	5.69	0.62	-
**37**	Spiraeoside	$C_{21}H_{20}O_{12}$	464.0952	5.77	-	7.57
**38**	Luteolin 7-O-glucoside (Cynaroside)	$C_{21}H_{20}O_{11}$	448.1002	6.26	-	0.90
**39**	Kaempferol-3-O-glucoside (Astragalin)	$C_{21}H_{20}O_{11}$	448.1005	6.59	-	35.61
**40**	Tricetin	$C_{15}H_{10}O_7$	302.0425	6.77	-	1.75
**41**	Quercitrin	$C_{21}H_{20}O_{11}$	448.1014	6.78	-	0.27
**42**	3,6,2',4'-Tetrahydroxyflavone	$C_{15}H_{10}O_6$	286.0484	6.78	-	0.02

**43**	Myricetin	$C_{15}H_{10}O_8$	318.0376	6.84	-	0.79
**44**	Resveratrol	$C_{14}H_{12}O_3$	228.0773	6.94	0.24	-
**45**	Rosmarinic acid	$C_{18}H_{16}O_8$	360.0852	7.24	2.22	-
**45**	Kaempferol-7-O-glucoside	$C_{21}H_{20}O_{11}$	448.1005	7.25	-	0.09
**47**	4-Methylumbelliferone	$C_{10}H_8O_3$	176.0476	7.42	21.32	-
**48**	Apigenin-7-O-glucoside (Apigetrin)	$C_{21}H_{20}O_{10}$	432.1054	7.47	-	2.05
**49**	Isohumulone	$C_{21}H_{30}O_5$	362.2140	7.82	0.78	-
**50**	Quercetin	$C_{15}H_{10}O_7$	302.0424	8.50	-	1.06
**51**	Tiliroside	$C_{30}H_{26}O_{13}$	594.1356	8.8	0.35	-
**52**	Cinnamic acid	$C_9H_8O_2$	148.0525	9.15	0.09	-
**53**	Capsaicin	$C_{18}H_{27}NO_3$	305.1999	9.66	0.21	-
**54**	Kaempferol	$C_{15}H_{10}O6$	286.0474	10.03	-	0.09
**55**	Diosmetin	$C_{16}H_{12}O_6$	300.0629	10.67	-	0.03
**56**	5,6,4'-Trihydroxy-7,3'-dimethoxyflavone	$C_{17}H_{14}O_7$	329.0663	11.02	-	0.02
**57**	3-Oxocostusic acid	$C_{15}H_{20}O_3$	248.1407	11.95	0.17	-
**58**	Madecassic acid	$C_{30}H_{48}O_6$	504.3442	14.35	0.24	-
**59**	Kumatakenin	$C_{17}H_{14}O6$	314.0788	14.38	-	0.10
**Total Compounds Identified**					99.77	99.62

Figure S3: LC-MS chromatograms of *A. andrachne* leaf extracts and identified compounds.

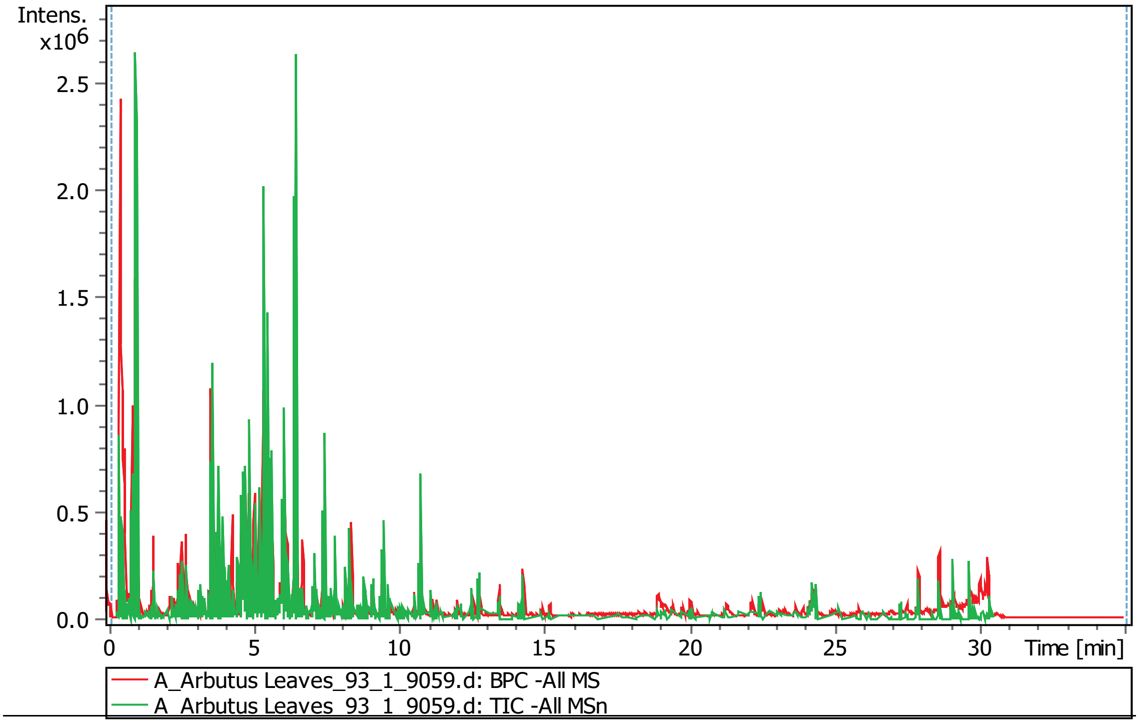

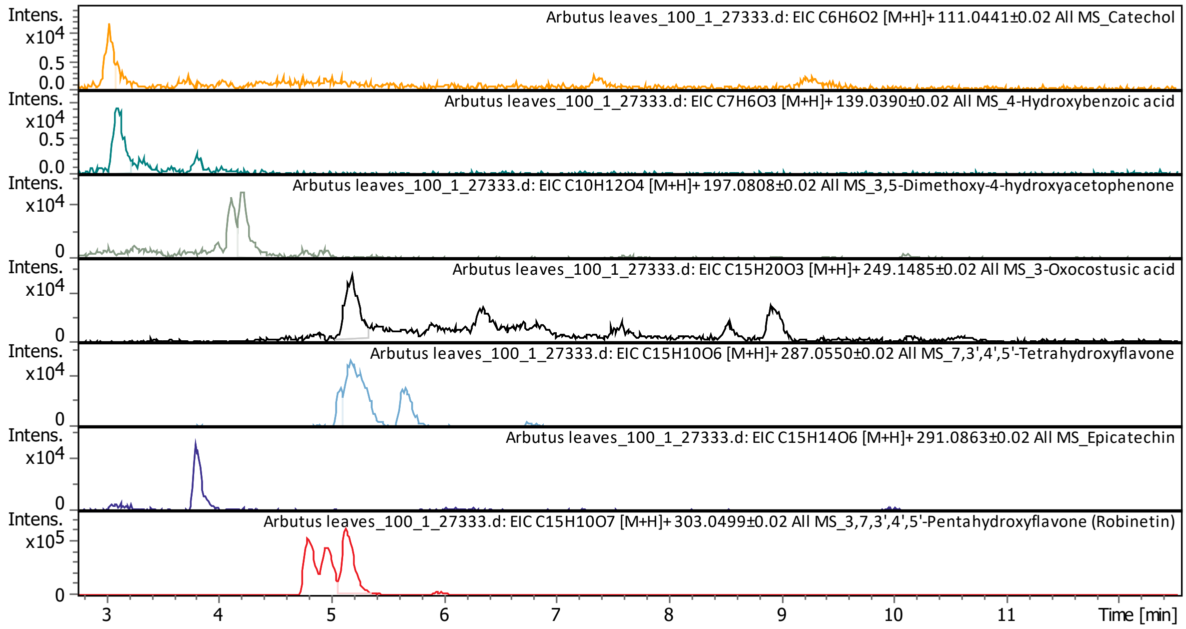

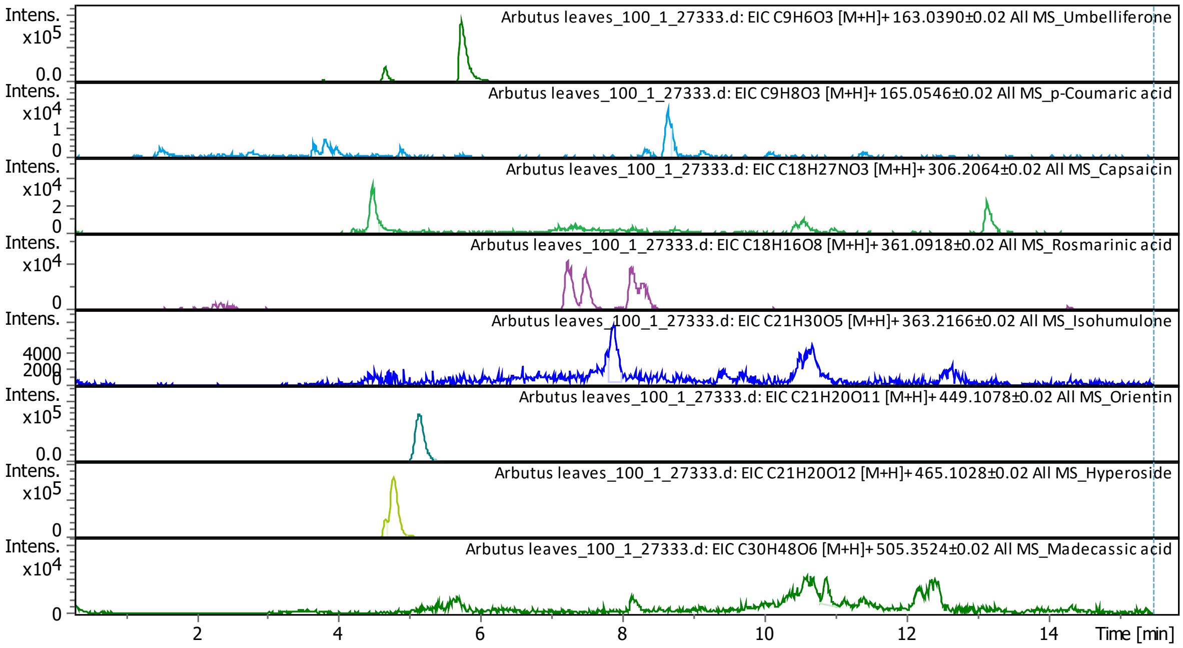
Arbutus leaves_100_1_27333.d: EIC C9H6O3 [M+H]+ 163.0390±0.02 All MS_Umbelliferone
Arbutus leaves_100_1_27333.d: EIC C9H8O3 [M+H]+ 165.0546±0.02 All MS_p-Coumaric acid
Arbutus leaves_100_1_27333.d: EIC C18H27NO3 [M+H]+ 306.2064±0.02 All MS_Capsaicin
Arbutus leaves_100_1_27333.d: EIC C18H16O8 [M+H]+ 361.0918±0.02 All MS_Rosmarinic acid
Arbutus leaves_100_1_27333.d: EIC C21H30O5 [M+H]+ 363.2166±0.02 All MS_Isohumulone
Arbutus leaves_100_1_27333.d: EIC C21H20O11 [M+H]+ 449.1078±0.02 All MS_Orientin
Arbutus leaves_100_1_27333.d: EIC C21H20O12 [M+H]+ 465.1028±0.02 All MS_Hyperoside
Arbutus leaves_100_1_27333.d: EIC C30H48O6 [M+H]+ 505.3524±0.02 All MS_Madecassic acid
Intens.
x10^5
x10^4
Time [min]

Figure S4: LC-MS chromatograms of *A. andrachne* fruit extracts.

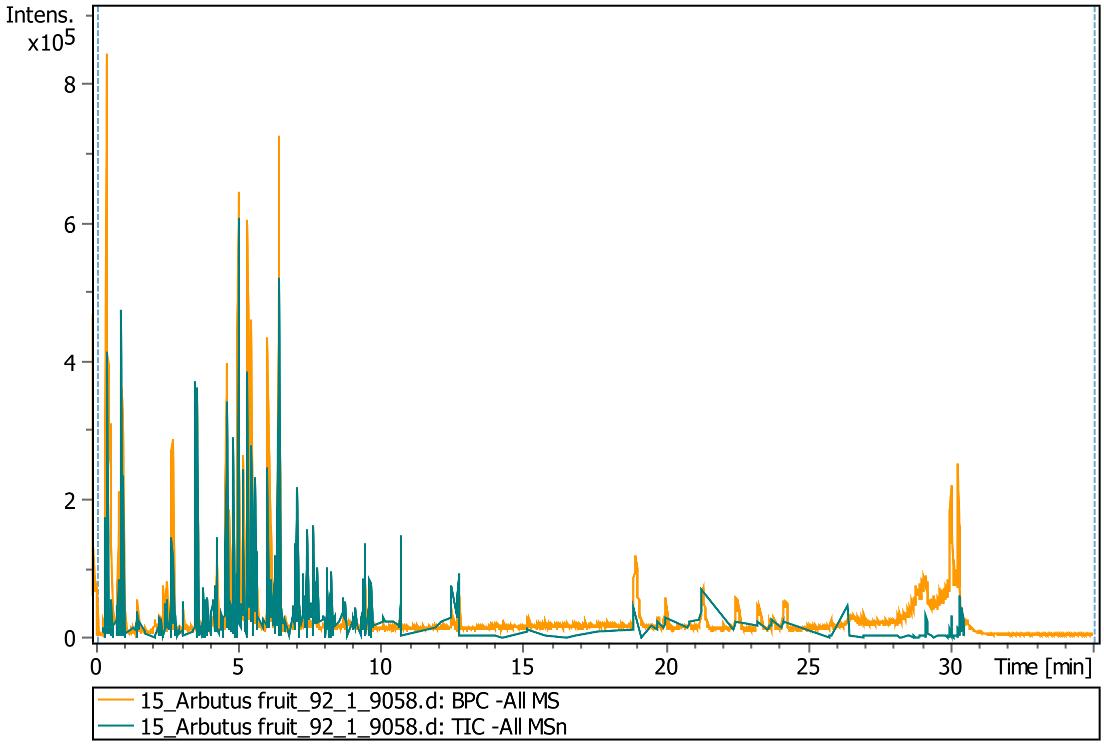

Supplement: Supplementary file 1 [file life-16-00560-s001.zip › life-4187151-supplementary.pdf]
